# Binge-watching addiction as an emotion regulation way of coping loneliness

**DOI:** 10.1371/journal.pone.0329853

**Published:** 2026-01-21

**Authors:** Xiaofan Yue, Xin Cui

**Affiliations:** 1 Huangshan University, Huangshan City, Anhui Province, China; 2 Huangshan University, Huangshan City, Anhui Province, China; Shandong University, CHINA

## Abstract

This study advances understanding of problematic media use by differentiating addictive from non-addictive binge-watching in relation to loneliness—an aspect often overlooked in prior research. It contributes to the field in three key ways: first, by empirically distinguishing problematic from non-problematic binge-watching; second, by demonstrating that loneliness significantly predicts binge-watching addiction but not non-problematic viewing; and third, by identifying escapism and emotional enhancement as dual emotion regulation pathways mediating this relationship, thereby extending existing single-motive frameworks. Using a sample of 551 participants, the study employed correlation and regression analyses to investigate these associations. Binge-watching addiction was assessed using the Problematic Series Watching Scale, and binge-watching motives were measured using the Watching TV Series Motives Questionnaire. Results revealed that loneliness was positively related to binge-watching addiction (*β* = 0.325, *p* < 0.01), but not to non-problematic binge-watching behavior. Analysis identified both escapism (*β* = 0.575, *p* < 0.01) and emotional enhancement motives (*β* = 0.429, *p* < 0.01) as significant factors contributing to binge-watching addiction, suggesting a dual-pathway model of emotion regulation. These findings refine theoretical models of maladaptive media use by illustrating how binge-watching addiction may serve as a behavioral emotion regulation strategy for coping with loneliness via both negative reinforcement and positive enhancement.

## Introduction

The outbreak of the COVID-19 pandemic has instigated notable changes in people’s lifestyles, leading to a significant uptick in sedentary indoor activities, such as binge-watching TV series [1]. This shift has sparked concerns regarding its potential relationships with mental health problems, particularly amid prolonged lockdown measures and social isolation [2]. The shift towards increased indoor activities during the pandemic has been linked to a range of mental health concerns, particularly as it intersects with prolonged periods of social isolation and emotional distress. However, there remains a limited understanding of the long-term psychological effects of these behaviors, especially in relation to binge-watching. Despite the increasing research attention on binge-watching behavior, our understanding of its effects remains limited, underscoring the need for a more thorough investigation into its association with mental health outcomes [3]. Although binge-watching has garnered significant research attention, key questions remain unanswered, particularly regarding its causal relationship with mental health issues. While some studies indicate a correlation between loneliness and depression, the exact nature of this relationship is still not well-understood, revealing a clear gap in the existing literature.

Of particular interest is the link between binge-watching and loneliness, a sentiment exacerbated by extended periods of social isolation. Previous studies have identified significant positive correlations between binge-watching and conditions such as depression, lack of self-regulation, and loneliness [4]. Among the various mental health outcomes, the relationship between binge-watching and loneliness has been particularly contentious. The causality of this relationship remains ambiguous, as some evidence suggests that loneliness may precede binge-watching behavior rather than arise from it [5].

The existing body of literature presents conflicting findings regarding the nexus between binge-watching and loneliness [6]. While certain studies have reported a positive correlation [7,8], others have yielded contradictory results [9–11]. These conflicting findings highlight the inadequacies in current research methods, which often fail to account for variables such as individual coping mechanisms, personality traits, and motivations for binge-watching. Moreover, these inconsistencies could arise from the lack of differentiation between occasional binge-watching and binge-watching addiction, which may have distinct psychological and emotional impacts on individuals. This gap in differentiating between problematic and non-problematic binge-watching has hindered a clearer understanding of how loneliness specifically influences various binge-watching behaviors.

These disparities underscore the need for a more nuanced investigation into the association between loneliness and binge-watching. Such inconsistencies may stem from factors such as concurrent conditions like depression and low self-control, which could influence the interplay between binge-watching and loneliness [12]. Another reason that may explain contradictory results is that the previous research has often overlooked crucial distinctions between occasional binge-watchers and individuals with binge-watching addiction [13]. By not adequately distinguishing between these two types of binge-watchers, prior research has failed to capture the complex psychological dynamics that may underlie the relationship between loneliness and binge-watching behavior. This gap in research has likely contributed to an incomplete understanding of how loneliness impacts binge-watching [14,15].

In light of these gaps, the principal aim of this study is to deepen our understanding of the complex relationship between feelings of loneliness and binge-watching, taking into account distinctions between non-problematic binge-watching and binge-watching addiction, as well as other contributing factors such as binge-watching motives. To address this gap, the present study makes several key contributions. First, it offers a clear empirical distinction between addictive and non-addictive binge-watching behaviors, a differentiation largely absent in previous research, thereby enabling a more nuanced understanding of how varying viewing patterns relate to psychological factors such as loneliness. Second, it establishes that loneliness significantly predicts binge-watching addiction but not non-problematic binge-watching, clarifying its role as a selective risk factor in problematic media use—an aspect often conflated in existing studies. Third, it identifies escapism and emotional enhancement as primary mediators of this relationship, thereby uncovering dual emotion regulation pathways that underlie problematic viewing behaviors, which have received limited attention in prior literature.

Additionally, we aim to explore the psychological motivations behind binge-watching to determine whether it functions as a form of emotional regulation. The novelty of our research lies in this dual-pathway approach to understanding binge-watching addiction, where we simultaneously examine both negative reinforcement (escapism) and positive enhancement motivations as potential mechanisms through which loneliness may lead to problematic viewing patterns.

## Literature review

### When Binge-watching turns into an addiction

Binge-watching is characterized as extended periods of focused, deliberate consumption of consecutive television episodes, typically narrative, suspenseful, and dramatic [16]. However, a precise definition of binge watchers remains elusive in current literature. Sung et al. [7] categorized individuals as high, medium, or low binge-watchers based on factors like episode count, viewing duration, and frequency. Some authors argue that both consecutive episode count [17] and session duration [18] should be considered in defining binge-watching, while others [19] suggest that sessions exceeding 4 hours qualify as problematic. Conversely, some studies do not stipulate a minimum duration [7,17]. Yet, a recent study [20] posited that quantifiable indicators, like episode count and viewing hours, may not reliably signify problematic binge-watching.

Previous studies have attempted to establish various thresholds for problematic binge-watching, with researchers increasingly recognizing that viewing duration alone is insufficient as a criterion. Research has evolved from purely quantitative measures to incorporating psychological and behavioral indicators in identifying problematic viewing patterns. This shift reflects a growing understanding that the context and consequences of viewing behavior are as important as the duration in determining its problematic nature.

The absence of a clear definition of binge-watching raises a crucial question: how do we differentiate between enthusiastic TV series viewing and problematic binge-watching? This distinction is vital for assessing whether the psychological underpinnings of problematic internet use apply to binge-watching, thus averting the risk of excessive pathologization of this leisure activity.

Current conceptualizations of binge-watching often blur the line between healthy leisure and problematic addictive behaviors. While the term “binge” connotes excessive activity devoid of self-control, binge-watching, unlike binge-eating or binge-drinking, may not inherently pose harm, depending on its impact on an individual’s life and its interference with other responsibilities [21]. Binge-watching is associated with the concept of “flow”, characterized by deep concentration, loss of self-awareness, distorted time perception, intrinsic enjoyment, and gratification [22]. Thus, binge-watching can be considered a healthy leisure pursuit until it spirals out of control, encroaching upon daily tasks like work or study.

Previous research has extensively examined the psychological mechanisms underlying flow states during binge-watching, revealing both positive and negative aspects of immersive viewing experiences. In the present study, when distinguishing between problematic binge-watching and non-problematic behavior, we characterize binge-watching addiction as a behavioral addiction characterized by exhibiting cravings, neglect of other activities, loss of control, tolerance, withdrawal symptoms, and adverse consequences [23]. Binge-watchers who did not score high on the binge-watching addiction measurement were considered to exhibit non-problematic binge-watching behavior. Unlike previous studies that have predominantly treated binge-watching as a homogeneous behavior, our research addresses a significant gap by establishing clear criteria to differentiate between problematic and non-problematic viewing patterns, allowing for more precise examination of their distinct relationships with psychological factors such as loneliness.

### Loneliness as antecedent of binge-watching

Loneliness, typified by feelings of social detachment and a dearth of meaningful connections, often drives individuals to seek solace in compensatory consumption, serving as a coping mechanism to alleviate the emotional void [24]. Research has demonstrated that media consumption patterns often intensify during periods of emotional distress, with several studies specifically linking increased streaming consumption to feelings of social isolation [4]. While this relationship has been documented across various demographic groups, the mechanisms underlying this connection remain complex and multifaceted. Considering loneliness as a predictor of problematic internet usage [25] and its documented association with problematic binge-watching in several studies [26,27], it is hypothesized that:

*H1. Loneliness is positively related to binge-watching addiction.*

Building upon conflicting findings regarding the association between loneliness and binge-watching, where some studies suggest no significant link [10–12], it is hypothesized that:

*H2. Loneliness is not related to non-problematic binge-watching.*

### Behavioral emotion regulation

Emotion regulation plays a pivotal role in various psychopathological conditions, including addiction [28], suggesting that the pursuit of mood regulation might play a significant role in the emergence of problematic binge-watching, and binge-watching has been suggested as a strategy for regulating emotions. [29]. Previous studies have identified various emotional regulation strategies employed during media consumption, including distraction, mood management, and social compensation [30,31]. Research has shown that individuals often consciously use media content to modify their emotional states, though the effectiveness and long-term implications of this strategy remain under debate.

Emotional regulation potentially acts as a mediating factor between loneliness and binge-watching addiction. A study by Gabbiadini et al. [27] underscored the significance of escapism in binge-watching, which fosters greater binge-watching inclinations. Escaping reality and experiencing transportation [31] are recognized as principal psychological factors driving binge-watching as a coping mechanism for loneliness. Binge-watching offers transient relief from negative emotions by diverting individuals’ attention from their feelings of loneliness and providing an avenue for escape [32].

Research has shown that the relationship between emotional regulation and binge-watching is particularly complex in the context of loneliness. Studies have revealed that individuals may simultaneously seek both emotional escape and emotional enhancement through binge-watching, suggesting a dual-process model of media consumption for emotional regulation [15].

Escapism, manifested through the suppression of loneliness emotions, represents one negative reinforcement motivation for coping with loneliness and is closely linked to binge-watching tendencies [27]. It is posited to be the primary factor in modeling problematic engagement in binge-watching. Therefore, it is hypothesized that:

*H3. Loneliness is related to escapism motive for binge-watching addiction.*

When binge-watching addiction is viewed as a Behavioral Emotion Regulation (BER) strategy for loneliness, escapism, characterized by avoidance and distraction, represents only one aspect of the Behavioral Emotion Regulation Questionnaire (BERQ) [31]. The other two scales of the BERQ—seeking social support and actively approaching—suggest that BER strategies encompass both negative reinforcement and positive enhancement motivations. Positive enhancement motivation involves seeking activities or experiences that enhance positive emotions and contribute to well-being. In contrast to escapism, which focuses on evading negative emotions, individuals driven by positive enhancement motivation actively strive to improve their mood and overall well-being through meaningful and enjoyable activities [32].

Previous research has identified distinct patterns in how individuals utilize different emotional regulation strategies through media consumption. Studies have found that while some viewers primarily seek escape from negative emotions, others actively pursue content that promotes positive emotional states, suggesting that individual differences in emotional regulation preferences may influence binge-watching behavior. Despite the growing body of literature on emotional regulation in media consumption, studies have often focused on isolated motivational pathways, leaving a critical gap in understanding how these pathways interact in the context of problematic viewing behaviors [33]. Our study addresses this gap by simultaneously examining both negative reinforcement (escapism) and positive enhancement motivations, offering a novel dual-pathway model that provides a more integrated view of emotion regulation in binge-watching addiction.

Additionally, The pursuit of entertainment and enjoyment, which are widely recognized as catalysts for engaging in recreational pursuits, are fundamental motivational elements in binge-watching [21]. Classic hedonic entertainment theories [34] propose that utilizing media is an efficient way to encounter enjoyable experiences, thereby evoking positive emotional states while reducing negative ones in media users [35]. While enhancing viewers’ enjoyment and hedonic well-being [36,37], binge-watching fits within the framework of behavioral emotion regulation (BER), aiming to alleviate mental health conditions such as loneliness. Therefore, we hypothesize that:

*H4. Loneliness is related to the emotional enhancement motive for binge-watching addiction.*

## Materials and methods

### Participants and procedures

After a thorough review by the Ethics Review Committee of Huangshan University, the study design and protocol were meticulously crafted to adhere to principles of safety and fairness, ensuring that the research content posed no harm or risk to the subjects involved. The study targeted Chinese adults aged 18 and above who regularly engage in watching TV series through various streaming platforms. The specific inclusion criteria required participants to be regular binge-watchers, defined as individuals who watched TV series for at least 3.5 consecutive hours and more than 4 episodes in one sitting during the week prior to the survey. All participants were adults, and recruitment was based on the principles of voluntary participation and informed consent. Recruitment was conducted through online channels, including social media platforms and streaming service user communities. In the study, participants who agreed to the informed consent proceeded to the next stage of the study, ensuring their voluntary participation. As all participants were required to acknowledge their consent before continuing with the survey, written consent was not deemed necessary.

To safeguard participant privacy, the research process and data collection were conducted anonymously. Prior to the commencement of the study, participants provided full informed consent. They were informed that the study would take approximately 5–15 minutes to complete. Participants were assured that their responses would be used solely for academic purposes and that they could withdraw from the study at any time without consequences. Data were collected via the Chinese survey web system, Wenjuanxing, during March 1–5th, 2024. Wenjuanxing provided sample service at the cost of 2 RMB per participant. We provided screening criteria, and Wenjuanxing selected qualified participants from their pool of over 6.2 million registered members to distribute the questionnaire. The exact amount of compensation participants received is unknown to us.

In the pre-screening survey, measures were taken to ensure that all participants met the criteria for binge-watchers. The target population consisted of individuals who regularly stream content through major platforms such as iQiyi, Youku, Tencent Video, and other popular streaming services in China. The engagement scale from the Binge-Watching Engagement and Symptoms Questionnaire (BWESQ) [15] and the definition of binge-watching [38] were utilized. Only individuals who answered affirmatively to the question, “Do you watch TV series for over 3.5 hours and more than 4 episodes in one sitting during the past week, and consider yourself to spend a significant amount of time watching TV series?” were included. This criterion was specifically chosen to align with contemporary definitions of binge-watching behavior in the Chinese context.

A total of 594 questionnaires were received. The participants represented diverse demographic backgrounds, including students, working professionals, and other adults from various regions across China. After excluding 34 respondents who answered negatively to the screening question, 560 questionnaires remained. To ensure the reliability of the sample and to identify participants who may not have been fully attentive, an attention check item (“Please answer 3 to this question”) was included. This attention check was strategically placed in the middle of the survey to maximize its effectiveness. Respondents who failed to answer “3” and whose response time was less than 3 minutes were excluded. Consequently, 551 valid questionnaires were retained for analysis. The final sample consisted of 551 Chinese binge-watchers (247 males [44.83%] and 304 females [55.17%]), with ages ranging from 18 to over 50 years old. Based on our addiction criteria using the Problematic Series Watching Scale, we divided participants into two groups: 217 in Sample 1 (without addiction; 46.08% male, mean age 28.6 years) and 334 in Sample 2 (with addiction; 44.01% male, mean age 27.9 years). Both samples were predominantly college-educated (67.74%), employed as company staff (49.77%), and represented a diverse cross-section of Chinese binge-watchers, with Sample 2 reporting higher binge-watching frequency.

### Measures

The questionnaire comprises two main sections. The first section gathers fundamental demographic data, encompassing gender, age, educational attainment, employment status, and frequency of engaging in binge-watching (defined as watching TV series for over 3.5 hours and more than 4 episodes in one sitting). The second section assesses various constructs using specific scales:

Measurement of binge-watching addiction was conducted using the Problematic Series Watching Scale questionnaire (PSWS) [39]. This scale consists of six items and is founded on the theoretical components model of addiction [40], aligning with the principal criteria of addiction from the DSM-5 pertaining to binge-watching. These criteria encompass salience (series watching dominates the individual’s thoughts), tolerance (where more series watching is needed to achieve previous effects), mood alteration (watching series to change one’s emotional state), relapse (returning to prior patterns of series watching despite trying to control it), withdrawal (feeling discomfort when unable to watch series), and conflict (series watching has negative impacts on relationships, work, or other aspects of life). Responses were recorded using a 5-point scale (1 = Never; 5 = Always), and all items were aggregated. The scale demonstrated excellent internal consistency reliability (Cronbach’s α = .906). For our research subjects, this scale was particularly appropriate as it enabled us to identify addiction patterns specifically among regular binge-watchers who may experience problematic symptoms related to their TV series consumption. As per the Bergen Work Addiction Scale [41], which sets addiction criteria as scoring “often” or “always” on at least 4 of the 7 items, we adopted a similar criterion [42]. The cut-off criterion for binge-watching addiction was established following the validated methodology of the Bergen Work Addiction Scale [41], which defines addiction based on scoring “often” or “always” on at least 4 of 7 items. This polythetic approach is consistent with established diagnostic frameworks for behavioral addictions in the DSM-5 and has been validated in previous studies of behavioral addictions [42]. We adapted this criterion for the 6-item PSWS while maintaining the same proportional threshold – requiring “often” or “always” responses on at least 4 of 6 items. Given that each item is scored on a 5-point scale (1 = Never to 5 = Always), and considering that scores of 4 (often) or 5 (always) on at least 4 items constitute the minimum threshold for addiction, this translates to a cut-off score of above 18 (4 items × 4 points + 2 items × 1 point = 18). Therefore, participants scoring above 18 were classified as having binge-watching addiction, while those scoring 18 or lower were categorized as non-problematic binge-watchers. This approach ensures theoretical consistency with established addiction criteria while maintaining methodological rigor in differentiating between problematic and non-problematic viewing patterns.

To evaluate escapism and emotional enhancement motivations, the Watching TV Series Motives Questionnaire (WTSMQ) [15] was utilized. The WTSMQ is a 22-item scale that assesses four main motivations for TV series watching: social, emotional enhancement, enrichment, and escapism. Each motivation is measured by 4–7 items. Respondents rated statements on a 4-point Likert scale, ranging from 1 (strongly disagree) to 4 (strongly agree). Both the escapism (Cronbach’s α = .948) and emotional enhancement (Cronbach’s α = .919) subscales demonstrated excellent internal consistency reliability.

The 6-Item UCLA Loneliness Scale (ULS-6) [43] was utilized. The ULS-6 is an abbreviated version of the original 20-item UCLA Loneliness Scale, designed to measure subjective feelings of loneliness and social isolation. Two reversed items (Item 3: I am an outgoing person; Item 6: I can find companionship when I want it) were excluded from the ULS-8, and the ULS-6 scale, which excludes these two items, was used in this study to ensure better psychometric properties and measurement accuracy. Participants provided their responses using a 4-point Likert scale, with options ranging from “never” to “always,” wherein higher scores denoted heightened levels of loneliness. The scale demonstrated excellent internal consistency reliability (Cronbach’s α = .937).

### Data analysis process

Descriptive statistics were calculated to summarize the data, followed by hypothesis testing to evaluate the significance of the trends observed. The data collected from the questionnaires was analyzed using SPSS. Initially, the data was cleaned, and any missing values (<1%) were handled using listwise deletion. The analysis process involved multiple stages to ensure a comprehensive examination of the research hypotheses.

First, reliability analyses were performed using Cronbach’s alpha to assess the internal consistency of all measurement scales. Second, descriptive statistics were computed to examine the distribution characteristics of the variables and summarize participant demographics. For hypothesis testing, the analysis was conducted separately for Sample 1 (non-addictive binge-watchers) and Sample 2 (addictive binge-watchers).

For Sample 1, linear regression analysis was conducted to examine the relationship between loneliness and binge-watching behavior to test H2. For Sample 2, Pearson correlation analyses were first conducted to examine the bivariate relationships between loneliness, binge-watching addiction, escapism, and emotional enhancement motives. Subsequently, linear regression analyses were performed to evaluate the relationships between variables and test H1, H3, and H4.

The assumptions of linear regression, including normality, linearity, and homoscedasticity, were verified through residual analysis. Multicollinearity was assessed using Variance Inflation Factors (VIF), with values below 5 considered acceptable. The significance level was set at p < 0.05 for all statistical tests, and standardized coefficients (beta) were reported to facilitate the interpretation of effect sizes.

## Results

To examine the relationships between key variables, correlation analyses were conducted using Sample 2 (n = 334, binge-watchers with addiction). This sample was chosen for correlation analysis as it represents individuals exhibiting problematic viewing patterns, allowing us to better understand the interrelationships between loneliness, binge-watching addiction, and viewing motives in this population. Initial analyses focused on examining correlations among key variables within Sample 2 (binge-watchers with addiction), followed by regression analyses to test specific hypotheses across both samples.

### Basic information of participants

As shown in Table 1, the analysis comprised valid responses from 551 participants who engaged in binge-watching behavior in the last week. Applying the cut-off criteria for binge-watching addiction, we identified 217 respondents scoring equal to or lower than 18 on PWAS, indicating an absence of addiction. These individuals are categorized as Sample 1 (binge-watchers without addiction), while Sample 2 consists of 334 participants classified as binge-watchers with addiction.

**Table 1:** Descriptive statistics.

		Sample 1 (Binge-watchers without addiction)		Sample 2 (Binge-watchers with addiction)	
Variable	Items	N	Percentage (%)	N	Percentage (%)
Gender	Male	100	46.08	147	44.01
	Female	117	53.92	187	55.99
Age	18–25	40	18.43	84	25.15
	21–30	44	20.28	89	26.65
	31–40	45	20.74	61	18.26
	41–50	36	16.59	64	19.16
	Over 50	52	23.96	36	10.78
Education	High school and below	50	23.04	37	11.08
	College	82	37.79	101	30.24
	University	65	29.95	166	49.70
	Master and above	20	9.22	30	8.98
Occupation	Student	12	5.53	32	9.58
	company staff	108	49.77	173	51.80
	Government official	33	15.21	40	11.98
	Teacher	20	9.22	36	10.78
	Others	44	20.28	53	15.87
Frequency of binge-watching	1-2 times per month	76	35.02		
	3-4 times per month	101	46.54		
	5-6 times per month	40	18.43	121	36.23
	Over 6 times per month			213	63.77
Total		217	100	334	100

In Sample 1, 100 males (46.08%) were represented, and participants span various age groups, predominantly holding college or university education (67.74%). The majority of participants were employed as company staff (49.77). Binge-watching frequency varied, with 81.56% reporting 1–4 times per month and 18.43% reporting 5–6 times per month. Sample 2 comprised 334 participants, with 147 males (44.01%) and 187 females (55.99%). Age, education, and occupation distributions mirrored those of Sample 1. However, binge-watching frequency was higher, with 36.23% reporting 5–6 times per month and 63.77% reporting over 6 times per month.

### Correlation analysis

Sample 2 (binge-watchers with addiction), comprising 334 respondents who meet the cut-off criteria for binge-watching addiction, underwent screening. This sample was chosen for correlation analysis as it represents individuals exhibiting problematic viewing patterns, allowing us to better understand the interrelationships between loneliness, binge-watching addiction, and viewing motives in this population.

The Pearson correlation matrix presented in Table 2, based on Sample 2 (binge-watchers with addiction, n = 334), demonstrates the relationships between the variables binge-watching addiction, loneliness, emotional enhancement, and escapism. These relationships directly address our core research purpose of differentiating how loneliness specifically influences problematic versus non-problematic binge-watching through distinct psychological pathways, revealing a more nuanced understanding than previous studies that treated binge-watching as a homogeneous behavior. The correlation patterns provide empirical support for our proposed dual-pathway model of emotion regulation in binge-watching addiction. The correlation coefficient between binge-watching addiction and loneliness is 0.325, which is statistically significant at the 0.01 level (p < 0.01). This indicates a significant positive correlation between binge-watching addiction and loneliness. This moderate correlation aligns with our theoretical framework, suggesting that loneliness acts as a psychological trigger for addictive viewing patterns. The correlation coefficient between binge-watching addiction and emotional enhancement motive is 0.370, also significant at the 0.01 level (p < 0.01), indicating a significant positive correlation between binge-watching addiction and emotional enhancement. The stronger correlation with emotional enhancement compared to loneliness suggests that the pursuit of positive emotions plays a crucial role in the development of problematic viewing patterns. The correlation coefficient between binge-watching addiction and escapism is 0.399, again significant at the 0.01 level (p < 0.01), indicating a significant positive correlation between binge-watching addiction and escapism. The strongest correlation with escapism supports previous research indicating that avoidance-based coping strategies are particularly relevant to the development of addictive behaviors.

**Table 2:** Pearson correlation coefficient matrix for Sample 2 (binge-watchers with addiction, n = 334).

	Binge-watching addiction	Loneliness	Emotional enhancement motive	Escapism motive
Binge-watching addiction	1			
Loneliness	0.325^**^	1		
Emotional enhancement motive	0.370^**^	0.429^**^	1	
Escapism motive	0.399^**^	0.575^**^	0.530^**^	1

*p < 0.05

**p < 0.01.

In summary, the correlation analysis reveals that binge-watching addiction is positively correlated with loneliness, emotional enhancement, and escapism. These findings suggest that individuals experiencing higher levels of loneliness, seeking emotional fulfillment, or using binge-watching as an escape mechanism are more likely to exhibit binge-watching addiction.

### Regression analyses

Following the correlation analyses, we conducted a series of regression analyses to test our hypotheses. First, we examined the relationship between loneliness and binge-watching behavior in Sample 1 (non-addictive binge-watchers) to test H2. Then, we conducted regression analyses using Sample 2 to test H1, H3, and H4, examining the relationships between loneliness, binge-watching addiction, and viewing motives.

As presented in Table 3, in Sample 1 (binge-watchers without addiction), a linear regression analysis was conducted to explore the relationship between loneliness and binge-watching addiction measurement. The analysis revealed a model formula of Addiction measurement = 2.294-0.076* Loneliness, with an R-squared value of 0.004, indicating that loneliness explains only 0.4% of the variance in binge-watching addiction measurement. However, the F-test results indicated a failure to pass the F-test (*F* = 0.838, *p* = 0.361 > 0.05), suggesting that loneliness does not significantly predict binge-watching addiction measurement. Hence, there is no significant relationship between the independent variable (loneliness) and the dependent variable (binge-watching addiction measurement). Thus, H2 (Loneliness is not related to non-problematic binge-watching) is supported.

**Table 3:** Linear regression analysis results for binge-watching without addiction predicted by loneliness in Sample 1 (n = 217).

Parameter Estimates (n = 217)							
	Unstandardized Coefficients		Standardized Coefficients	*t*	*p*	Collinearity diagnostics	
	Beta	Std. Error	Beta		VIF	Tolerance	
Constant	2.294	0.221	–	10.363	0.000^**^	–	–
Loneliness	−0.076	0.083	−0.062	−0.915	0.361	1.000	1.000
*R* ^2^	0.004						
Adj *R* ^2^	−0.001						
*F*	*F* (1,215)=0.838, *p* = 0.361						

Dependent Variable: Binge-watching without addiction.

* p < 0.05

** p < 0.01.

Table 4 presents the results of a linear regression analysis, with loneliness as the independent variable and predicting binge-watching addiction. The analysis revealed that loneliness was the sole variable retained in the final model, with an R-squared value of 0.106, indicating that approximately 10.6% of the variance in binge-watching addiction could be attributed to loneliness. The F-test result (*F* = 39.211, *p* = 0.000) indicates the model’s statistical significance, suggesting that loneliness significantly influences binge-watching addiction. Further examination of the model’s parameters indicates that the regression coefficient for loneliness is 0.329, with a corresponding t-value of 6.262 (*p* = 0.000 < 0.01), indicating a statistically significant positive relationship between loneliness and binge-watching addiction. In essence, higher levels of loneliness are associated with increased levels of binge-watching addiction. Moreover, Diagnostic examinations for multicollinearity indicated that all Variance Inflation Factor (VIF) values fell below 5, indicating the lack of multicollinearity concerns. Therefore, the analysis underscores that loneliness significantly predicts binge-watching addiction, emphasizing its noteworthy positive impact on binge-watching behavior. Thus, H1. Loneliness is positively related to binge-watching addiction is supported. This finding is particularly significant as it empirically demonstrates that loneliness is significantly associated with addictive viewing patterns, rather than general binge-watching. This underscores the importance of differentiating between problematic and non-problematic viewing behaviors in both research and clinical contexts. The moderate effect size (β = 0.325) suggests that loneliness may play a notable role in problematic media consumption, and addressing it could serve as a potential intervention point for reducing such behaviors. However, further research is needed to determine whether loneliness acts as a specific trigger or operates in conjunction with other factors in driving addictive viewing patterns.

**Table 4:** Linear regression analysis results for binge-watching addiction predicted by loneliness in Sample 2 (n = 334).

Parameter Estimates (*n* = 334)							
	Unstandardized Coefficients		Standardized Coefficients	*t*	*p*	Collinearity diagnostics	
	Beta	Std. Error	Beta		VIF	Tolerance	
Constant	3.143	0.185	–	16.978	0.000^**^	–	–
Loneliness	0.329	0.053	0.325	6.262	0.000^**^	1.000	1.000
*R* ^2^	0.106						
Adj *R* ^2^	0.103						
*F*	*F* (1,332)=39.211, *p* = 0.000						

Dependent Variable: Binge-watching addiction measurement.

* p < 0.05

** p < 0.01.

Continuing with Sample 2 (binge-watchers with addiction, n = 334), as shown in Table 5, loneliness was selected as the independent variable, while escapism was treated as the dependent variable. The model demonstrated statistical significance with an R-squared value of 0.331 and passed the F-test, indicating its effectiveness. The regression equation for the model is Escapism = 1.537 + 0.554** loneliness. Additionally, an assessment of multicollinearity showed that all VIF values were less than 5, indicating no multicollinearity concerns. In conclusion, the regression coefficient for loneliness (β = 0.575, *t* = 12.809, *p* = 0.000 < 0.01) implies a notable affirmative correlation between loneliness and escapism. Therefore, H3 (Loneliness is related to escapism motive for binge-watching addiction) is supported.

**Table 5:** Linear regression analysis results for escapism predicted by loneliness in Sample 2 (n = 334).

Parameter Estimates (*n* = 334)							
	Unstandardized Coefficients		Standardized Coefficients	*t*	*p*	Collinearity diagnostics	
	Beta	Std. Error	Beta		VIF	Tolerance	
Constant	1.537	0.152	–	10.092	0.000^**^	–	–
Loneliness	0.554	0.043	0.575	12.809	0.000^**^	1.000	1.000
*R* ^2^	0.331						
Adj *R* ^2^	0.329						
*F*	*F* (1,332)=164.082, *p* = 0.000						

Dependent Variable: Escapism

*p < 0.05

**p < 0.01.

As shown in Table 6, using Sample 2 (binge-watchers with addiction, n = 334), after conducting regression analysis with loneliness as the independent variable and enhancement as the dependent variable, the model automatically retained loneliness as the only predictor. This suggests that loneliness can explain 18.4% of the variance in emotional enhancement, with an R-squared value of 0.184. Furthermore, the model passed the F-test, indicating its statistical significance. The regression equation derived from the model is Enhancement = 1.983 + 0.435* loneliness. All VIF values were under 5, suggesting the lack of multicollinearity problems. In conclusion, the regression coefficient for loneliness (β = 0.429, t = 8.654, p = 0.000 < 0.05) signifies a significant positive relationship between loneliness and emotional enhancement. Thus, it can be summarized that loneliness has a significant positive impact on the emotional enhancement motive for binge-watching. Therefore, H4 (Loneliness is related to emotional enhancement motive for binge-watching addiction) is supported.

**Table 6:** Linear regression analysis results for emotional enhancement predicted by loneliness in Sample 2 (n = 334).

Parameter Estimates (*n* = 334)							
	Unstandardized Coefficients		Standardized Coefficients	*t*	*p*	Collinearity diagnostics	
	Beta	Std. Error	Beta		VIF	Tolerance	
Constant	1.983	0.177	–	11.202	0.000^**^	–	–
Loneliness	0.435	0.050	0.429	8.654	0.000^**^	1.000	1.000
*R* ^2^	0.184						
Adj *R* ^2^	0.182						
*F*	*F* (1,332)=74.888,*p* = 0.000						

Dependent Variable: Emotional Enhancement.

*p < 0.05

**p < 0.01.

### Mediation analyses

Following the regression analyses, we conducted a series of mediation analyses to further examine the mechanisms underlying the relationships between loneliness and binge-watching addiction. Specifically, we explored the potential mediating roles of escapism and emotional enhancement in Sample 2. The mediation models tested the extent to which these emotional regulation pathways account for the relationship between loneliness and binge-watching addiction.

As shown in Table 7, the mediation analysis explored the impact of loneliness on binge-watching addiction through the mediator escapism. The results show that the total effect of loneliness on binge-watching addiction was significant (c = 0.329, p < 0.01). The mediation effect through escapism (a * b = 0.184, p < 0.01) was also significant. After including the mediator escapism, the direct effect (c’ = 0.145, p < 0.05) was reduced but remained significant. The mediation effect through escapism accounted for approximately 56.03% of the total effect (a * b/ c = 56.031%). While loneliness continues to directly affect binge-watching addiction, the mediation through escapism explains a substantial portion of the relationship.

**Table 7:** Mediation effect test for the relationship between loneliness, escapism, and binge-watching addiction in Sample 2 (n = 334).

Variable	Total Effect (c)	a	b	Mediation Effect (a^*^b)	Boot SE (a^*^b)	z Value (a^*^b)	p Value (a^*^b)	95% Boot CI (a^*^b)	Direct Effect (c’)	Conclusion
LO => ES => AD	0.329^**^	0.554^**^	0.333^**^	0.184	0.072	2.565	0.010	0.037 ~ 0.316	0.145^*^	Partial Mediation

Note:

*p < 0.05,

**p < 0.01.

LO = Loneliness, ES = Escapism, AD = Binge-watching Addiction.

Bootstrap method = Percentile Bootstrap.

Table 8 demonstrates that the mediation analysis explored the impacts of loneliness on binge-watching addiction through the mediator emotional enhancement. The results indicate that the total effect of loneliness on binge-watching addiction was significant (c = 0.329, p < 0.01). The mediation effect through emotional enhancement (a * b = 0.123, p < 0.01) was also significant. After including the mediator emotional enhancement, the direct effect (c’ = 0.207, p < 0.01) was reduced but remained significant. These findings suggest that emotional enhancement partially mediates the relationship between loneliness and binge-watching addiction, accounting for approximately 37.27% of the total effect (a * b/ c = 37.268%). While loneliness continues to directly affect binge-watching addiction, the mediation through emotional enhancement plays a substantial role in explaining how loneliness contributes to binge-watching behavior.

**Table 8:** Mediation Effect Test for the Relationship between Loneliness, Emotional Enhancement, and Binge-watching Addiction in Sample 2 (n = 334).

Variable	Total Effect (c)	a	b	Mediation Effect (a^*^b)	Boot SE (a^*^b)	z Value (a^*^b)	p Value (a^*^b)	95% Boot CI (a^*^b)	Direct Effect (c’)	Conclusion
LO=> EE=> AD	0.329^**^	0.435^**^	0.282^**^	0.123	0.053	2.314	0.021	0.019 ~ 0.227	0.207^**^	Partial Mediation

Note:

*p < 0.05,

**p < 0.01.

LO = Loneliness, EE = Emotional Enhancement, AD = Binge-watching Addiction.

Bootstrap method = Percentile Bootstrap.

According to Table 9, the mediation analysis explored the impact of loneliness on binge-watching addiction through the mediators escapism and emotional enhancement. The results showed that the total effect of loneliness on binge-watching addiction was significant (c = 0.329, p < 0.01). Both mediators, escapism (a * b = 0.135, p = 0.018) and emotional enhancement (a * b = 0.088, p = 0.027), significantly mediated the relationship. After including the mediators, the direct effect (c’ = 0.107, p < 0.01) was reduced but remained significant.

**Table 9:** Mediation effect test for the relationship between loneliness, escapism, emotional enhancement, and binge-watching addiction in Sample 2 (n = 334).

Variable	Total Effect (c)	a	b	Mediation Effect (a^*^b)	Boot SE (a^*^b)	z Value (a^*^b)	p Value (a^*^b)	95% Boot CI (a^*^b)	Direct Effect (c’)	Conclusion
LO=>ES=>AD	0.329^**^	0.554^**^	0.243^**^	0.135	0.057	2.364	0.018	0.024 ~ 0.247	0.107	Full Mediation
LO=>EE=>AD	0.329^**^	0.435^**^	0.202^**^	0.088	0.040	2.214	0.027	0.014 ~ 0.169	0.107	Full Mediation

Note:

*p < 0.05,

**p < 0.01.

LO = Loneliness, ES = Escapism, EE = Emotional Enhancement, AD = Binge-watching Addiction.

Bootstrap method = Percentile Bootstrap.

These findings suggest that when escapism and emotional enhancement are analyzed together as part of a dual-path emotional regulation model, they fully mediate the relationship between loneliness and binge-watching addiction. This means that the effect of loneliness on binge-watching addiction is entirely transmitted through these two emotional regulation pathways, with no direct effect remaining. Consequently, the direct relationship between loneliness and binge-watching addiction becomes non-significant, with all effects transmitted through escapism and emotional enhancement.

## Discussion

The study’s results illuminate the intricate relationship between loneliness, binge-watching addiction, and binge-watching motivations. These findings not only address a critical gap in existing literature regarding the differential effects of loneliness on problematic versus non-problematic media consumption patterns, but also advance the field by establishing a novel dual-pathway model of emotion regulation in problematic viewing. Our empirical demonstration that loneliness specifically predicts addictive but not non-problematic binge-watching challenges prevailing assumptions in media psychology and addiction studies that have often treated intensive media use as inherently problematic without considering the underlying psychological mechanisms. These findings address a critical gap in existing literature regarding the differential effects of loneliness on problematic versus non-problematic media consumption patterns. Our primary findings suggest that loneliness is not significantly associated with non-problematic binge-watching, indicating that individuals engaging in binge-watching without addictive tendencies may not do so as a means of coping with loneliness. This distinction is particularly important as it challenges the common assumption that all forms of intensive media consumption are driven by psychological distress. Instead, our results suggest that the relationship between loneliness and binge-watching is more nuanced, with problematic patterns emerging specifically when viewing behavior is motivated by emotional regulation needs. This nuanced understanding of the loneliness-binge-watching relationship has important implications for both the theoretical conceptualization of media addiction and the development of practical intervention approaches. Theoretically, it suggests that problematic media use should be distinguished from non-problematic intensive use, as the underlying psychological mechanisms may differ. In terms of interventions, it highlights the need to target the specific emotional regulation deficits that contribute to the development of addictive viewing patterns.

Regression analyses offered valuable insights into the correlation between loneliness and binge-watching addiction. The statistical approach employed in this study, using linear regression, was specifically designed to address the primary research question of whether loneliness serves as a significant predictor of problematic viewing patterns. By modeling the direct effect of loneliness on binge-watching addiction, we were able to quantify the strength of this relationship while controlling for potential confounding variables. Loneliness positively predicted binge-watching addiction, aligning with prior hypotheses and supporting existing literature linking loneliness to problematic internet use [44]. This relationship demonstrated through a robust regression coefficient (β = 0.325, p < 0.01), provides empirical evidence for the theoretical framework that emotional states can drive addictive media consumption patterns. The magnitude of this coefficient suggests a moderate to strong effect size, indicating that loneliness explains a substantial portion of the variance in binge-watching addiction scores. This validates the association between problematic binge-watching and loneliness [26,45,46]. The strength of this relationship suggests that loneliness may be a key risk factor in the development of problematic viewing behaviors, particularly in contexts where alternative social connections are limited. It suggests that previous studies which found no link between loneliness and binge-watching [10–12] may have focused on non-problematic binge-watching, devoid of addiction symptoms. This methodological distinction between problematic and non-problematic viewing highlights the importance of considering viewing patterns along a continuum from healthy to problematic, rather than treating all intensive viewing as potentially problematic. By differentiating between these patterns, our study provides a more precise understanding of the psychological factors that contribute to the development of media addiction. This underscores the emerging concept that problematic binge-watching may reflect maladaptive coping strategies [29,47,48], necessitating differentiation from non-problematic binge-watching. The identification of these distinct patterns contributes to the development of more nuanced theoretical models for understanding media addiction and may inform the development of targeted interventions for individuals at risk.

Following the regression analyses, the mediation analysis demonstrated that escapism and emotional enhancement fully mediate the relationship between loneliness and binge-watching addiction. Specifically, when analyzed together as part of a dual-path emotional regulation model, these mediators account entirely for the relationship, with no direct effect remaining. This indicates that loneliness influences binge-watching addiction primarily through these two emotional regulation mechanisms, where escapism serves as a means of avoiding negative emotions, and emotional enhancement functions as a strategy to pursue positive emotional states.

These findings underscore the complex nature of binge-watching addiction, suggesting that it stems not only from the avoidance of negative emotions (escapism) but also from the desire to enhance positive emotions (emotional enhancement). The dual-path model provides a more nuanced understanding of how loneliness contributes to binge-watching addiction, highlighting the roles of both avoidance-based and approach-based coping strategies in sustaining problematic viewing behaviors. The identification of these dual pathways stresses the importance of designing targeted interventions that address both escapism and emotional enhancement motivations. These results contribute to the theoretical understanding of media addiction by proposing that problematic binge-watching may arise from a combination of emotional needs, rather than a sole focus on negative reinforcement.

While affirming the positive relationship between loneliness and binge-watching addiction, our results also reveal the mediating roles of escapism and emotional enhancement motives. This finding addresses a key research objective of understanding the psychological mechanisms through which loneliness influences viewing behavior. By incorporating these motives as potential mediators, our analysis provides insight into the ways in which loneliness may lead to problematic viewing patterns. The significant associations between loneliness and both escapism and emotional enhancement suggest that these motives serve as important links in the causal chain from loneliness to addiction. These findings resonate with prior research, indicating that individuals may binge-watch to escape negative emotions or enhance positive ones [20]. The dual nature of these motivations, involving both avoidance of negative affect and pursuit of positive affect, suggests a complex interplay between avoidance-based and approach-based coping strategies in the context of media consumption. This highlights the need for theoretical models that can account for multiple, potentially competing, psychological processes in the development of problematic viewing behavior.

Incorporating escapism and emotional enhancement motives into the regression model alongside loneliness provided crucial insights**.** The improved model fit (R^2^ increase from 0.106 to 0.148) demonstrates the significant explanatory power added by considering these motivational factors. This increase suggests that a substantial portion of the variation in binge-watching addiction scores can be accounted for by the combination of loneliness and viewing motives. The fact that both motives remained significant predictors in the model, even when controlling for loneliness, indicates that they have unique explanatory power and are not merely proxies for loneliness. Both motives significantly contributed to binge-watching addiction, indicating that individuals with higher levels of these motives were more likely to exhibit addictive binge-watching behavior. This finding challenges the traditional view that problematic media use is primarily driven by escapism alone, revealing instead a more complex interaction where both negative reinforcement (escapism) and positive enhancement operate simultaneously as distinct yet complementary pathways from loneliness to addiction. This finding extends existing theoretical frameworks by suggesting that problematic binge-watching may develop through multiple pathways, each potentially requiring different intervention approaches. The dual-pathway model offers new possibilities for targeted interventions that address both avoidance-based (escapism) and approach-based (emotional enhancement) motivations in problematic viewing.

The relationship between loneliness and binge-watching motives, particularly escapism and emotional enhancement, sheds light on the behavioral and emotional dynamics of binge-watching addiction. This analysis directly addresses our research objective of understanding how binge-watching functions as an emotional regulation strategy. By examining the associations between loneliness and specific viewing motives, we gain insight into the ways in which individuals attempt to cope with feelings of social disconnection through media consumption. Significant associations between loneliness and these motives were evident in the regression coefficients: escapism motive = 1.537 + 0.554 * Loneliness and emotional enhancement motive = 1.983 + 0.435 * Loneliness. The stronger coefficient for escapism (0.554) compared to emotional enhancement (0.435) suggests that loneliness may more strongly drive avoidance-based coping strategies than approach-based ones. The stronger association between loneliness and escapism (β = 0.575) compared to emotional enhancement (β = 0.429) suggests a prioritization of negative reinforcement mechanisms in the development of problematic viewing patterns, adding important nuance to theoretical models of media addiction that have typically treated all motivations as equally influential. These findings highlight the complex relationship between loneliness and motives for binge-watching. This discovery reveals the previously unidentified hierarchical structure of viewing motivations among lonely individuals, offering a more precise framework for understanding the developmental trajectory from normal to problematic viewing behavior in the context of loneliness. The differential strength of these relationships, with escapism being more strongly associated with loneliness than emotional enhancement, provides novel insights into the hierarchical structure of viewing motivations among lonely individuals. This suggests that interventions aimed at reducing problematic binge-watching may need to prioritize targeting escapism motives, as they appear to be more central in the link between loneliness and addiction.

Positive coefficients indicate that individuals experiencing heightened levels of loneliness are more likely to endorse both escapism and emotional enhancement as motives for binge-watching. This dual-pathway model, involving both avoidance and approach motivations, represents an important theoretical advancement in understanding media addiction. It suggests that intervention strategies may need to address both negative reinforcement and positive enhancement motivations simultaneously, rather than focusing on a single motivational process. The identification of multiple, potentially complementary emotional regulation strategies expands our understanding of the psychological mechanisms underlying problematic binge-watching and provides a more comprehensive framework for conceptualizing media addiction. This suggests that binge-watching may function as a coping mechanism for individuals aiming to mitigate feelings of loneliness, either by escaping from negative emotions (escapism) or by enhancing positive ones (emotional enhancement). The identification of these distinct but related pathways contributes to a more nuanced understanding of how emotional regulation strategies may evolve into problematic viewing patterns. It suggests that the development of binge-watching addiction may involve a complex interplay between different coping mechanisms, each reinforcing the other in a self-perpetuating cycle. This insight has important implications for the design of interventions, as it suggests that targeting only one pathway (e.g., escapism) may be insufficient if the other pathway (e.g., emotional enhancement) continues to drive problematic behavior.

Moreover, these results align with the concept of Behavioral Emotion Regulation, which proposes that individuals may engage in specific behaviors to regulate their emotions. Within the context of binge-watching, escapism represents a form of negative reinforcement motivation, where individuals seek to alleviate loneliness by escaping from negative emotions or stressors. Conversely, emotional enhancement reflects a positive motivation, wherein individuals strive to enhance positive emotions or experiences through binge-watching. These findings support a previous study’s conclusions, which indicated that a propensity towards escapism during television series watching significantly influences problematic viewing engagement [20]. Negative reinforcement factors, such as managing loneliness or seeking escapism from daily worries, often underlie problematic engagement in various online activities [49]. Thus, our results suggest that binge-watching addiction functions as a multifaceted coping mechanism for loneliness, encompassing both negative reinforcement (escapism) and positive enhancement (emotional enhancement) motivations. This finding represents a significant theoretical contribution by challenging the traditional dichotomous view of media consumption motivations.

Understanding these nuanced motivations is crucial for developing targeted interventions to address problematic binge-watching behaviors and mitigate feelings of loneliness effectively. The identification of multiple concurrent pathways to problematic viewing suggests that intervention strategies may need to be more comprehensive than previously thought, addressing both avoidance and enhancement motivations simultaneously. Our findings contradict previous research suggesting that different motivations (positive reinforcement vs. negative reinforcement) lead to different outcomes (non-harmful vs. problematic involvement) in binge-watching [20]. This contradiction highlights the need to revisit existing theoretical models of media addiction that may oversimplify the relationship between viewing motivations and outcomes. One potential explanation for this inconsistency could be that binge-watching serves as a multifaceted strategy for emotion regulation, offering both an escape from negative feelings and a source of positive emotions. This dual-function hypothesis extends current theoretical frameworks by suggesting that the same behavioral pattern may simultaneously serve multiple psychological needs, complicating the traditional view of adaptive versus maladaptive media use. Furthermore, these findings suggest that the development of problematic viewing patterns may be more complex than previously understood, involving the interaction of multiple motivational systems rather than the dominance of a single pathway.

As the concept of binge-watching addiction as a strategy for regulating emotions becomes more recognized, a promising direction for enhancing our comprehension of binge-watching would be to examine the diverse array of emotion regulation tactics that viewers utilize during binge-watching. This research direction emerges naturally from our findings regarding the dual pathways of emotional regulation in problematic viewing behavior. The identification of multiple concurrent emotional regulation strategies suggests the need for more sophisticated theoretical models that can account for the complexity of viewer motivations and behaviors.

## Conclusion

We provide insights into the nuanced interplay between loneliness, binge-watching motives, and addiction, highlighting the multifaceted nature of binge-watching behavior as both a coping mechanism and a form of emotional regulation. This study makes several notable contributions. It clearly distinguishes between addictive and non-addictive binge-watching—an area often oversimplified in previous research. It further demonstrates that loneliness is significantly associated with addictive binge-watching, but not with non-addictive viewing behavior, thereby challenging previous assumptions that all binge-watching behaviors are driven by uniform psychological mechanisms. Finally, it introduces a dual-pathway emotion regulation model involving both escapism and emotional enhancement, offering a more comprehensive framework for understanding how loneliness influences binge-watching addiction compared to prior studies that emphasized singular motivational pathways. This research directly addresses our primary aim of understanding how loneliness specifically influences different types of binge-watching behaviors by empirically demonstrating that loneliness predicts binge-watching addiction (*β* = 0.325, *p* < 0.01) but not non-problematic viewing patterns. Furthermore, we successfully identified the dual emotional regulation pathways through which lonely individuals engage in problematic viewing, fulfilling our objective to explore the psychological mechanisms behind binge-watching. First, this research establishes a comprehensive empirical framework for understanding how loneliness contributes to the development of problematic viewing patterns through multiple motivational pathways. Second, it demonstrates the importance of considering both negative and positive reinforcement mechanisms in the development of media addiction. We encourage researchers to reassess binge-watching’s association with mental health outcomes, based on our distinction between addictive and non-addictive patterns. This distinction represents a crucial advancement in our theoretical understanding of media addiction, suggesting that the pathway from normal to problematic viewing behavior may be mediated by specific psychological vulnerabilities and motivational factors. The identification of these distinct pathways has important implications for both prevention and intervention strategies, suggesting the need for targeted approaches that address the underlying psychological mechanisms rather than focusing solely on viewing behavior.

Our findings are in line with the BER framework, demonstrating how individuals utilize binge-watching to regulate their negative emotions, particularly in coping with loneliness. This alignment with established theoretical frameworks provides important validation for our findings while also extending our understanding of how emotional regulation mechanisms operate in the context of digital media consumption. The successful application of the BER framework to binge-watching suggests that other theoretical models from the broader field of addiction and emotional regulation research may also be relevant to understanding problematic viewing patterns. We contribute to the understanding of binge-watching motives by elucidating the distinct roles of negative reinforcement and positive reinforcement motivations in influencing binge-watching addiction. This dual-pathway model represents a significant theoretical advancement, suggesting that the development of problematic viewing patterns involves a more complex interplay of motivational factors than previously recognized. Furthermore, our findings suggest that future theoretical models of media addiction should consider how different motivational systems may interact and reinforce each other, potentially creating self-sustaining cycles of problematic use.

Our empirical investigation aimed to deepen our understanding of the complex relationship between loneliness and binge-watching, taking into account the distinctions between non-problematic and addictive binge-watching behaviors, as well as the contributing factors of binge-watching motives. Our findings directly answer the study’s central questions by confirming that loneliness is positively associated with binge-watching addiction but not with non-problematic binge-watching. Moreover, our analysis revealed that both escapism (*β *= 0.575, *p* < 0.01) and emotional enhancement motives (*β* = 0.429, *p* < 0.01) significantly contribute to binge-watching addiction among lonely individuals, supporting our hypothesis that binge-watching functions as a multifaceted emotion regulation strategy. These results suggest that binge-watching serves as a multifaceted emotion regulation strategy for coping with loneliness, encompassing both negative reinforcement (escapism) and positive enhancement (emotional enhancement) motivations.

Our study offers several notable strengths, including a comprehensive empirical framework, clear differentiation between problematic and non-problematic viewing patterns, and the successful application of the BER framework to the context of binge-watching. However, we acknowledge certain limitations. One important limitation is the scope of our definition of binge-watching behavior, which adhered to the traditional conceptualization of consuming consecutive TV episodes. This definition does not encompass the growing concerns surrounding problematic video streaming behaviors on platforms such as YouTube and TikTok [50], where continuous consumption of short-form content may also constitute a form of binge-watching. Moreover, problematic live-streaming, as observed in studies by Chaudhary et al. [51] and Yang et al. [52], has gained attention as a potential area of concern. A survey by Cabeza-Ramírez et al. [53] found that 6.4% of video game players (n = 37/580) reported problematic video game streaming. The inclusion of these diverse forms of content consumption may provide a more comprehensive understanding of binge-watching, particularly given the shorter, non-consecutive nature of content on modern streaming platforms. This limitation in scope raises important questions about how evolving media consumption patterns might influence the relationship between loneliness and problematic viewing behaviors. As the streaming landscape continues to diversify, it may be necessary to expand theoretical frameworks to account for these new forms of media engagement.

Further investigation into cultural and contextual variations in binge-watching behavior could enhance our understanding of the phenomenon, considering how societal norms and technological affordances impact individuals’ motivations and binge-watching patterns. Our findings from the Chinese context provide a foundation for cross-cultural comparisons, particularly regarding how different societies view and engage with streaming media. The strong relationship we found between loneliness and binge-watching addiction in Chinese viewers raises important questions about whether this pattern holds true across different cultural contexts where social connections and media consumption habits may vary significantly. Additionally, studying binge-watchers’ loneliness levels after prolonged viewing sessions could deepen our understanding of the relationship between binge-watching and loneliness. This suggestion emerges directly from our findings about the dual pathways of emotional regulation, as we discovered that both escapism and emotional enhancement motives play significant roles in viewing behavior. Understanding the temporal dynamics of these relationships could reveal whether binge-watching provides effective short-term emotional relief but potentially exacerbates loneliness in the long term. Given the limitations of cross-sectional design, future qualitative studies could offer in-depth insights into the subjective experiences and motivations underlying binge-watching behavior, complementing quantitative findings and providing a more comprehensive understanding of individuals’ binge-watching experiences.

## Supporting information

S1 Data Current Data- eng. (XLSX)

## References

[1] Rahman KT, Arif MZ. Impacts of binge-watching on Netflix during the COVID-19 pandemic. South Asian Journal of Marketing. 2021;2(1):97–112.

[2] West K. Unsurprising: Netflix survey indicates people like to binge-watch TV. Cinema-Blend. http://www.cinemablend.com/television/Unsurprising-Netflix-Survey-Indicates-People-Like-Binge-Watch-TV-61045.html. 2014.

[3] Joseph D, Varghese SK. Binge-watching as a behavioural addiction: A systematic review of causes, consequences, and potential for mental health intervention. Indian J Psychiatr Soc Work. 2025;36:36–47.

[4] Starosta JA, Izydorczyk B. Understanding the Phenomenon of Binge-Watching-A Systematic Review. Int J Environ Res Public Health. 2020;17(12):4469. doi: 10.3390/ijerph17124469 32580289 PMC7344932

[5] Alfonsi V, Varallo G, Scarpelli S, Gorgoni M, Filosa M, De Gennaro L, et al. “This is the last episode”: the association between problematic binge-watching and loneliness, emotion regulation, and sleep-related factors in poor sleepers. J Sleep Res. 2023;32(1):e13747. doi: 10.1111/jsr.13747 36254098 PMC10078456

[6] Rutsch P. Does binge-watching make us depressed? Good question. NPR. http://www.npr.org/sections/health-shots/2015/02/04/383527370/does-binge-watching-make-us-depressed-goodquestion. 2015.

[7] Wingralek ZJ, Banaszek A, Giermasinski A, Czarnecki A, Goliszek K, Kajka N, et al. Turn on the screen, turn off the loneliness-analysis of risk factors for binge-watching among Polish medical and non-medical students. A web-based cross-sectional study. Ann Agric Environ Med. 2024;31(2). 10.26444/aaem/183779 38940111

[8] Yu H, Alizadeh F. Online binge-watching among chinese college students: implications for loneliness, anxiety, and depression. Psychology Research and Behavior Management. 2024:295–303. 38292254 10.2147/PRBM.S447311 PMC10826546

[9] Tokunaga RS, Rains SA. An evaluation of two characterizations of the relationships between problematic Internet use, time spent using the Internet, and psychosocial problems. Human Communication Research. 2010;36(4):512–45.

[10] Tukachinsky R, Eyal K. The Psychology of Marathon Television Viewing: Antecedents and Viewer Involvement. Mass Communication and Society. 2018;21(3):275–95. doi: 10.1080/15205436.2017.1422765

[11] Tefertiller AC, Maxwell LC. Depression, emotional states, and the experience of binge-watching narrative television. Atlantic Journal of Communication. 2018;26(5):278–90.

[12] Ahmed AA-AM. A new era of tv-watching behavior: binge watching and its psychological effects. Media Watch. 2017;8(2). doi: 10.15655/mw/2017/v8i2/49006

[13] Özdemir Y, Kuzucu Y, Ak Ş. Depression, loneliness and internet addiction: how important is low self-control? Computers in Human Behavior. 2014;34:284–90.

[14] Forte G, Favieri F, Tedeschi D, Casagrande M. Binge-watching: development and validation of the binge-watching addiction questionnaire. Behav Sci (Basel). 2021;11(2):27. doi: 10.3390/bs11020027 33672253 PMC7926824

[15] Flayelle M, Canale N, Vögele C, Karila L, Maurage P, Billieux J. Assessing binge-watching behaviors: Development and validation of the “Watching TV Series Motives” and “Binge-watching Engagement and Symptoms” questionnaires. Computers in Human Behavior. 2019;90:26–36.

[16] Alimoradi Z, Jafari E, Potenza MN, Lin CY, Wu CY, Pakpour AH. Binge-watching and mental health problems: a systematic review and meta-analysis. Int J Environ Res Public Health. 2022;19(15):9707. 35955069 10.3390/ijerph19159707 PMC9368441

[17] Schweidel DA, Moe WW. Binge watching and advertising. Journal of Marketing. 2016;80(5):1–19.

[18] Trouleau W, Ashkan A, Ding W, Eriksson B. Just one more: modeling binge watching behavior. In: Proceedings of the 22nd ACM SIGKDD International Conference on Knowledge Discovery and Data Mining, 2016:1215–24.

[19] Vaterlaus JM, Spruance LA, Frantz K, Kruger JS. College student television binge watching: Conceptualization, gratifications, and perceived consequences. The Social Science Journal. 2019;56(4):470–9. doi: 10.1016/j.soscij.2018.10.004

[20] Flayelle M, Elhai JD, Maurage P, Vögele C, Brevers D, Baggio S, et al. Identifying the psychological processes delineating non-harmful from problematic binge-watching: A machine learning analytical approach. Telematics and Informatics. 2022;74:101880.

[21] Vallerand RJ, Blanchard C, Mageau GA, Koestner R, Ratelle C, Leonard M, et al. Les passions de l’ame: on obsessive and harmonious passion. J Pers Soc Psychol. 2003;85(4):756–67. doi: 10.1037/0022-3514.85.4.756 14561128

[22] Ilyas U, Maqsood R, Ahsan H, Rauf A, Afzal S. Binge-watching as behavioral addiction: a systematic review. APR. 2023;2(2):39–65. doi: 10.32350/apr.22.03

[23] Starcevic V. Is Internet addiction a useful concept? Aust N Z J Psychiatry. 2013;47(1):16–9. doi: 10.1177/0004867412461693 23293309

[24] Rippé CB, Smith B, Weisfeld-Spolter S. The connection of attachment and self-gifting for the disconnection of loneliness across cultures. Int J Consum Stud. 2022;46(4):1451–67.

[25] Orsolini L, Longo G, Volpe U. The mediatory role of the boredom and loneliness dimensions in the development of problematic internet use. Int J Environ Res Public Health. 2023;20(5):4446. doi: 10.3390/ijerph20054446 36901452 PMC10001960

[26] Sun J-J, Chang Y-J. Associations of problematic binge-watching with depression, social interaction anxiety, and loneliness. Int J Environ Res Public Health. 2021;18(3):1168. doi: 10.3390/ijerph18031168 33525732 PMC7908146

[27] Gabbiadini A, Baldissarri C, Valtorta RR, Durante F, Mari S. Loneliness, escapism, and identification with media characters: an exploration of the psychological factors underlying binge-watching tendency. Front Psychol. 2021;12:785970. doi: 10.3389/fpsyg.2021.785970 35069369 PMC8771202

[28] Kraft L, Ebner C, Leo K, Lindenberg K. Emotion regulation strategies and symptoms of depression, anxiety, aggression, and addiction in children and adolescents: A meta-analysis and systematic review. Clin Psychol Sci Pract. 2023;30(4):485.

[29] Flayelle M, Maurage P, Vögele C, Karila L, Billieux J. Time for a plot twist: Beyond confirmatory approaches to binge-watching research. Psychology of Popular Media Culture. 2019;8(3):308.

[30] Lutz S, Schneider FM, Reich S. Media as powerful coping tools to recover from social exclusion experiences? A systematic review on need restoration and emotion regulation through using media. Media Psychol. 2023;26(4):388–413.

[31] Kraaij V, Garnefski N. The behavioral emotion regulation questionnaire: development, psychometric properties and relationships with emotional problems and the cognitive emotion regulation questionnaire. Personality and Individual Differences. 2019;137:56–61.

[32] Fa’im Rosli NS, Mahudin ND. One more episode won’t hurt! How stress, social interaction anxiety, and loneliness relate to binge-watching behaviours among university students. J Commun Lang Cult. 2024;4(1):1–20.

[33] Flayelle M, Maurage P, Di Lorenzo KR, Vögele C, Gainsbury SM, Billieux J. Binge-watching: what do we know so far? a first systematic review of the evidence. Curr Addict Rep. 2020;7(1):44–60. doi: 10.1007/s40429-020-00299-8

[34] Reinecke L. Mood management theory. The International Encyclopedia of Media Effects. 2017:–3.

[35] Vorderer P, Reinecke L. From mood to meaning: The changing model of the user in entertainment research. Communication Theory. 2015;25(4):447–53.

[36] Granow VC, Reinecke L, Ziegele M. Binge-watching and psychological well-being: media use between lack of control and perceived autonomy. Communication Research Reports. 2018;35(5):392–401. doi: 10.1080/08824096.2018.1525347

[37] Halfmann A, Reinecke L. Binge-watching as case of escapist entertainment use. The Oxford handbook of entertainment theory. 2021:181–203.

[38] Aghababian AH, Sadler JR, Jansen E, Thapaliya G, Smith KR, Carnell S. Binge Watching during COVID-19: associations with stress and body weight. Nutrients. 2021;13(10):3418. doi: 10.3390/nu13103418 34684420 PMC8539795

[39] Orosz G, Bőthe B, Tóth-Király I. The development of the Problematic Series WatchingScale (PSWS). J Behav Addict. 2016;5(1):144–50. doi: 10.1556/2006.5.2016.011 28092185 PMC5322994

[40] Griffiths M. A ‘components’ model of addiction within a biopsychosocial framework. Journal of Substance Use. 2005;10(4):191–7.

[41] Andreassen CS, Griffiths MD, Hetland J, Pallesen S. Development of a work addiction scale. Scand J Psychol. 2012;53(3):265–72. doi: 10.1111/j.1467-9450.2012.00947.x 22490005

[42] Orosz G, Dombi E, Andreassen CS, Griffiths MD, Demetrovics Z. Analyzing models of work addiction: Single factor and bi-factor models of the Bergen Work Addiction Scale. Int J Mental Health and Addiction. 2016;14:662–71.

[43] Zhou L, Li Z, Hu M, Xiao S. Reliability and validity of ULS-8 loneliness scale in elderly samples in a rural community. Journal of Central South University Medical Sciences. 2012;37(11):1124–8. 23202622 10.3969/j.issn.1672-7347.2012.11.008

[44] Moretta T, Buodo G. Problematic internet use and loneliness: how complex is the relationship? a short literature review. Current Addiction Reports. 2020;7:125–36.

[45] Batik MV, Demir M. The mediating role of binge-watching in the relationship between type D personality and loneliness. Health Psychol Rep. 2021;10(3):157–67. doi: 10.5114/hpr.2021.109550 38084275 PMC10679904

[46] Raza SH, Yousaf M, Sohail F, Munawar R, Ogadimma EC, Siang JMLD. Investigating binge-watching adverse mental health outcomes during covid-19 pandemic: moderating role of screen time for web series using online streaming. Psychol Res Behav Manag. 2021;14:1615–29. doi: 10.2147/PRBM.S328416 34675702 PMC8518137

[47] Boursier V, Musetti A, Gioia F, Flayelle M, Billieux J, Schimmenti A. Is watching TV series an adaptive coping strategy during the COVID-19 pandemic? Insights from an Italian community sample. Frontiers in Psychiatry. 2021;12:599859. doi: 10.3389/fpsyt.2021.599859 33967845 PMC8097049

[48] Sigre-Leirós V, Billieux J, Mohr C, Maurage P, King DL, Schimmenti A, et al. Binge-watching in times of COVID-19: A longitudinal examination of changes in affect and TV series consumption patterns during lockdown. Psychology of Popular Media. 2023;12(2):173.

[49] Bowditch L, Chapman J, Naweed A. Do coping strategies moderate the relationship between escapism and negative gaming outcomes in World of Warcraft (MMORPG) players? Computers in Human Behavior. 2018;86:69–76.

[50] Rahat M, Mojgani J, Lethbridge G, Al-Bya H, Patterson B, Goldman Bergmann C, et al. Problematic video-streaming: a short review. Current Opinion in Behavioral Sciences. 2022;48:101232. doi: 10.1016/j.cobeha.2022.101232

[51] Chaudhary A, Saroha J, Monteiro K, Forbes AG, Parnami A. Are you still watching?: exploring unintended user behaviors and dark patterns on video streaming platforms. In: Proceedings of the 2022 ACM Designing Interactive Systems Conference, 2022:776–91.

[52] Yang Z, Griffiths MD, Yan Z, Xu W. Can watching online videos be addictive? a qualitative exploration of online video watching among chinese young adults. Int J Environ Res Public Health. 2021;18(14):7247. doi: 10.3390/ijerph18147247 34299696 PMC8306552

[53] Cabeza-Ramírez LJ, Muñoz-Fernández GA, Santos-Roldán L. Video game streaming in young people and teenagers: uptake, user groups, dangers, and opportunities. Healthcare (Basel). 2021;9(2):192. doi: 10.3390/healthcare9020192 33578675 PMC7916337

